# Integrative proteomic and functional analyses provide novel insights into the action of the repurposed drug candidate nitroxoline in AsPC-1 cells

**DOI:** 10.1038/s41598-020-59492-4

**Published:** 2020-02-13

**Authors:** Serena Veschi, Maurizio Ronci, Paola Lanuti, Laura De Lellis, Rosalba Florio, Giuseppina Bologna, Luca Scotti, Erminia Carletti, Federica Brugnoli, Maria Cristina Di Bella, Valeria Bertagnolo, Marco Marchisio, Alessandro Cama

**Affiliations:** 1 Department of Pharmacy, G. d’Annunzio University of Chieti-Pescara, Chieti, Italy; 2 Department of Medical, Oral and Biotechnological Sciences, G. d’Annunzio University of Chieti-Pescara, Chieti, Italy; 3 Centre on Aging Sciences and Translational Medicine (Ce.S.I-Me.T), G. d’Annunzio University of Chieti-Pescara, Chieti, Italy; 4 Department of Medicine and Aging Sciences, G. d’Annunzio University of Chieti-Pescara, Chieti, Italy; 5 Section of Anatomy and Histology, Department of Morphology, Surgery and Experimental Medicine, University of Ferrara, Ferrara, Italy

**Keywords:** Cell biology, Pancreatic cancer

## Abstract

We recently identified nitroxoline as a repurposed drug candidate in pancreatic cancer (PC) showing a dose-dependent antiproliferative activity in different PC cell lines. This antibiotic is effective in several *in vitro* and animal cancer models. To date, the mechanisms of nitroxoline anticancer action are largely unknown. Using shotgun proteomics we identified 363 proteins affected by nitroxoline treatment in AsPC-1 pancreatic cancer cells, including 81 consistently deregulated at both 24- and 48-hour treatment. These proteins previously unknown to be affected by nitroxoline were mostly downregulated and interconnected in a single highly-enriched network of protein-protein interactions. Integrative proteomic and functional analyses revealed nitroxoline-induced downregulation of Na/K-ATPase pump and β-catenin, which associated with drastic impairment in cell growth, migration, invasion, increased ROS production and induction of DNA damage response. Remarkably, nitroxoline induced a previously unknown deregulation of molecules with a critical role in cell bioenergetics, which resulted in mitochondrial depolarization. Our study also suggests that deregulation of cytosolic iron homeostasis and of co-translational targeting to membrane contribute to nitroxoline anticancer action. This study broadens our understanding of the mechanisms of nitroxoline action, showing that the drug modulates multiple proteins crucial in cancer biology and previously unknown to be affected by nitroxoline.

## Introduction

Pancreatic cancer (PC) is characterized by a short patient survival and a high lethality after diagnosis^1^. The poor prognosis of this severe disease is due to late diagnosis and to unsatisfactory response to current radiotherapy and chemotherapy regimens^2^. Therefore, more effective therapeutic approaches and less toxic drugs are needed for PC treatment. In this respect, it has been reported that several non-anticancer agents approved for the treatment of different human diseases have anticancer properties^3,4^. These drugs are excellent candidates to be repurposed in cancer therapy due to their potential for a rapid translation of preclinical findings in human therapy. In addition, considering that drug combinations are generally more effective than single agents^1^ and that drugs candidate for repurposing often show low toxicity, they could be used in combination, possibly with less side effects as compared to therapeutic protocols combining standard chemotherapeutic agents. Currently, we have a limited understanding of the mechanisms of action of repurposed drug candidates in cancer. Antitumor actions of these molecules appear to be related to multiple off-target effects^3,5^. In this regard, considering that several biological pathways are deregulated in tumors, drugs acting on multiple targets are thought to be more effective than single-target agents and might have a greater potential to circumvent drug resistance^6^. Overall, gaining a better insight into the mechanisms of action of repurposed drugs in tumors is crucial, because it could reveal proteins previously unknown to be relevant in cancer therapy and could help in crafting rational and synergistic combinations of drugs acting on complementary pathways.

In a recent study, we analyzed the effects of nitroxoline as a candidate for repurposing in pancreatic cancer, showing that the drug has antitumor effects comparable to erlotinib, a targeted drug approved for PC treatment^7^. In that study, nitroxoline was found to affect viability, to drastically reduce clonogenic activity, to hamper cell cycle and to promote apoptosis in pancreatic cancer cell lines, both as a single agent and in combinations with other drugs^7^. In addition to pancreatic cancer, this drug affects viability and growth of cell lines representative of other tumors, including neuroblastoma, glioma, myeloma, prostate, lung, kidney and bladder cancers^8–19^. Remarkably, this antibiotic is also effective in several cancer xenograft and genetically engineered mice models^8–10,12,14,15,18^. These findings indicate that nitroxoline is a promising candidate for repurposing in cancer treatment.

Nitroxoline is an antibiotic widely used in several countries from 1960s and is able to chelate divalent metal ions such as Mg^2+^ and Mn^2+^, an activity which is considered as a possible mechanism for its antibacterial action^3^. To analyze mechanisms by which this antibacterial exerts its anticancer activity, several studies screened for specific candidate targets and molecular pathways known to play an important role in cancer biology^7–19^. Using this approach, disparate targets have been proposed to be responsible for nitroxoline anticancer effects. However, studies designed to screen for specific candidate targets may provide a biased window of observation on the potential off-target effects of the drug and it is conceivable that some important target has been missed by candidate target approaches. Notably, unbiased approaches, such as proteomic studies, have the potential to reveal additional proteins and molecular pathways affected by nitroxoline. In this regard, studies analyzing the effects of nitroxoline on proteomic expression profiles in cancer cells are currently lacking.

In the present study, using a shotgun proteomic approach we analyzed the effect of nitroxoline on protein expression in AsPC-1, a metastatic pancreatic cancer cell line resistant to the first-line PC chemotherapy agent gemcitabine^20^, but sensitive to nitroxoline^7^. The results of this study show that the majority of proteins affected by nitroxoline treatment are interconnected in a highly enriched network of protein-protein interactions. In addition, this integrative study shows that nitroxoline modulates a number of proteins and pathways crucial in cancer biology, which were previously unknown to be deregulated by this drug.

## Results

### Shotgun proteomics identifies proteins consistently deregulated by nitroxoline

In this study, an isotope free shotgun proteomics approach was used to identify proteins differentially expressed in AsPC-1 cells treated with nitroxoline *versus* vehicle for 24 or 48 hours. Three biological replicates for each time point (24- and 48-hours) were prepared and each one was analyzed in 2–3 technical replicates. Thereafter, we used two levels of processing. The first had an FDR ≤0.5% at the peptide-spectrum matches (PSM) level for every single technical replicate, which resulted in a protein FDR <1%. A total of 1782 unique proteins were confidently identified at this level. The second level of processing was the label-free relative quantification to detect modulated proteins, which was performed through the label-free quantification module PEAKS-Q, part of PEAKS Studio 7.5. This quantification method is based on the relative areas of the extracted ion chromatograms of peptides detected in multiple samples and applies the expectation-maximization algorithm to detect and resolve overlapping features. High-performance retention time alignment algorithm was used to align the features of the same peptide from multiple samples^21^. The differentially expressed proteins were calculated for each biological replicate using 0.5% PSMs FDR and the resulting ID lists were filtered considering only peptides identified in at least 3 out of 6 technical replicates (untreated *vs*. treated), significance ≥20 (−10lgP), quality factor ≥0.5, Average Area ≥ 10^4^, and by considering only proteins identified with significance ≥20 and fold change ≥2. This generated a list of deregulated proteins for each biological replicate at 24- and 48-hour treatment with their relative expression ratios. These lists were manually curated by merging the lists of each replicate in a single file and removing keratins, resulting in 190 and 254 proteins for 24- and 48-hour treatment, respectively (Supplementary Table 1). For subsequent analyses we selected the 81 proteins identified and consistently deregulated at both time points (Supplementary Table 2). Most of these proteins were downregulated. Except for cathepsin B, which was downregulated only at 24 hours, all other proteins identified in this proteomic analysis were not previously known to be modulated by nitroxoline. Among them, ribosomal proteins and proteins related to metabolism or energy pathways were highly represented (Supplementary Table 2). Examples of these proteins are listed in Table 1.

**Table 1:** Examples of proteins consistently deregulated by nitroxoline at 24- and 48-hour treatment.

**Protein**	**Gene**	Ratio nitroxoline *vs*. vehicle control	
		**24 h**	**48 h**
**Up- regulated**			
Transferrin receptor protein 1	**TFRC**	3.74	5.88
Phosphoglycerate kinase 1	**PGK1**	2.47	5.26
Fructose-bisphosphate aldolase A	**ALDOA**	2.57	2.32
L-lactate dehydrogenase A chain (LDH-A)	**LDHA**	2.39	2.17
**Down- regulated**			
Citrate synthase, mitochondrial	**CS**	0.49	0.50
Fumarate hydratase, mitochondrial	**FH**	0.29	0.38
Poly(rC)-binding protein 1 (Alpha-CP1) (Heterogeneous nuclear ribonucleoprotein E1)	**PCBP1**	0.50	0.35
Cytochrome b-c1 complex subunit 1, mitochondrial	**UQCRC1**	0.42	0.34
Catenin beta-1 (Beta-catenin)	**CTNNB1**	0.35	0.32
Sodium/potassium-transporting ATPase subunit alpha-1 Na(+)/K(+)	**ATP1A1**	0.49	0.30
Cytochrome c oxidase subunit 2	**MT-CO2**	0.42	0.22
Cytochrome b-c1 complex subunit 2, mitochondrial	**UQCRC2**	0.36	0.19
Signal recognition particle 14 kDa protein	**SRP14**	0.30	0.16
Succinate-CoA ligase [ADP-forming] subunit beta, mitochondrial	**SUCLA2**	0.40	0.11
NADH dehydrogenase [ubiquinone] iron-sulfur protein 3, mitochondrial	**NDUFS3**	0.28	0.08
Sodium/potassium-transporting ATPase subunit beta-3 Na(+)/K(+)	**ATP1B3**	0.38	0.08
Aconitate hydratase, mitochondrial	**ACO2**	0.00	0.00

### Most proteins deregulated by nitroxoline are connected in a single functional network

**Figure 1:**
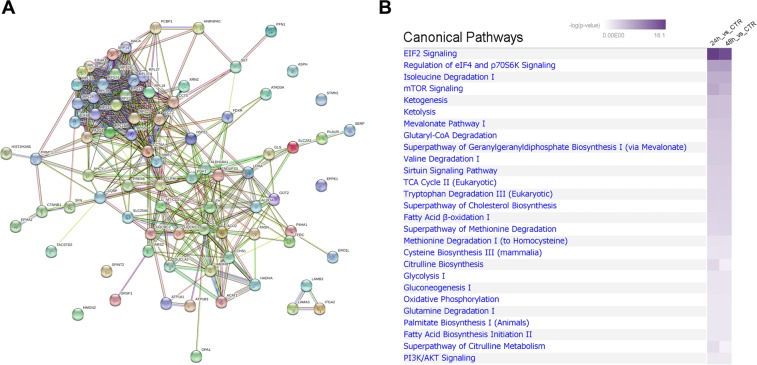
Predicted interactions among proteins consistently deregulated by nitroxoline and canonical pathways affected by the drug. (**A**) The figure shows interactions predicted by STRING analysis among 81 proteins consistently deregulated by nitroxoline at 24 and 48 hours. Apart from 5 proteins that showed no predicted interactions and additional 3 proteins forming a small interacting cluster, all the remaining proteins deregulated by nitroxoline were predicted to interact in a single, highly enriched, protein-protein interaction functional network (p-value < 10^−16^). (**B**) The highest ranked pathways according to IPA proved to be very similar at both time points. These included some of the pathways known to be modulated by nitroxoline, such as PI3K/AKT signaling, sirtuins and mTOR, as well as several pathways previously unknown to be deregulated by the drug.

The dataset of 81 proteins consistently deregulated at both time points was submitted to STRING, a database of known and predicted protein-protein interactions. Notably, the results of this analysis showed that the majority of the proteins (73 of the 81 submitted proteins) are connected within a single network, with a highly significant protein-protein interaction enrichment (p-value < 10^−16^) (Fig. 1A). We also tested whether STRING analysis would predict interactions among the 81 proteins consistently deregulated by nitroxoline and the proteins that were previously indicated to be affected by this drug^7,9,12–15,17–19^. Notably, these proteins previously known to be deregulated by nitroxoline are connected with the main functional interaction network of proteins identified in this study (Supplementary Fig. S1).

### Proteomic analysis identifies pathways previously unknown to be modulated by nitroxoline

The datasets of proteins deregulated at 24- or 48-hour treatment were then submitted to Ingenuity Pathway Analysis (IPA) for Gene Ontology to assess enriched canonical pathways and protein networks, followed by a Comparison Analysis between both time points. As shown in Fig. 1B, the highest ranked pathways were very similar at both time points and included some of the pathways previously known to be modulated by nitroxoline, such as PI3K/AKT signaling, sirtuins and mTOR^12,15,17^ (Fig. 1B). According to IPA, the highest ranked pathways were EIF2 signaling and regulation of eIF4 and p70S6K signaling, which were not known to be modulated by nitroxoline (Fig. 1B). The related pathways “translation” and “SRP-dependent cotranslational protein targeting to membrane” (REACTOME: HSA-72766 and HSA-1799339, respectively) resulted highly enriched (FDR 1.5 × 10^−17^ and 3.04 × 10^−18^, respectively) by STRING analysis (Supplementary Table 3) and proteins in this pathway were consistently downregulated by nitroxoline. In line with IPA and STRING analyses, we observed a significant decrease in cell surface protein labeling by flow cytometry after treatment with nitroxoline (Supplementary Fig. S2). Thus, proteomic and flow cytometry analyses indicate that protein synthesis and targeting to membrane pathways, which have a crucial role in cancer cell growth, are affected by nitroxoline. Among additional pathways critical for cancer biology and previously unknown to be deregulated by nitroxoline, several of the highest ranked according to IPA and STRING analyses were related to cell bioenergetics and metabolism, including tricarboxylic acid cycle (TCA), respiratory electron transport, aminoacid, lipid and carbohydrate metabolism (Fig. 1B and Supplementary Table 3 and Supplementary Table 4).

### Validation of proteins previously unknown to be affected by nitroxoline treatment

**Figure 2:**
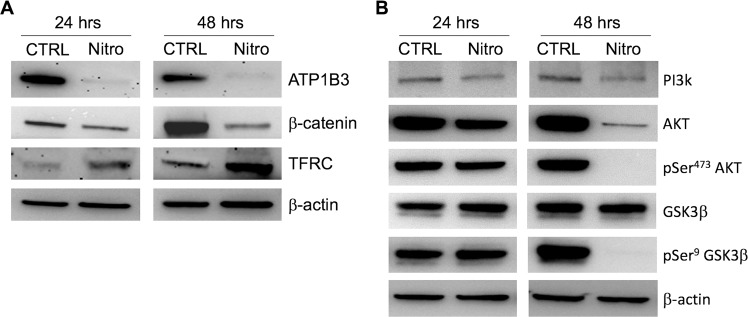
Western blot validation of proteins consistently deregulated by nitroxoline in proteomic analysis and effects of the treatment on PI3K/AKT/GSK3β/β-catenin signaling pathway. (**A**) Western blot analysis confirmed that nitroxoline induced shifts in ATP1B3, β-catenin and TFRC protein expression consistent with proteomic analysis. Full-length of these representative western blots are shown in Supplementary Fig. S3. (**B**) Representative western blots of indicated proteins in AsPC-1 cells treated with vehicle (control) or nitroxoline for 24 and 48 hours. The results are in line with the downregulation of PI3K/AKT/GSK3β/β-catenin pathway. Full-length of western blots are shown in Supplementary Fig. S4.

Among the 81 proteins consistently deregulated by nitroxoline treatment and relevant in cancer biology, three were selected for western blot validation, namely the ATP1B3 subunit of Na/K-ATPase pump, β-catenin (CTNNB1) and transferrin receptor protein 1 (TFRC) (Table 1 and Fig. 2A). Western blot analysis confirmed that ATP1B3 and β-catenin were consistently downregulated, while TFRC was markedly upregulated by nitroxoline treatment at both time points in AsPC-1. Since downregulation of ATP1B3 was previously shown to result in reduced expression of PI3K, AKT and pAKT in gastric cancer^22^ and these molecules are known to regulate β-catenin expression through phosphorylation of GSK3β^23^, we analyzed the possibility that this pathway was involved in β-catenin downregulation. Western blot analysis showed that nitroxoline-induced downregulation of ATP1B3 was associated with reduced expression of PI3K and AKT, as well as with reduced phosphorylation of AKT (pSer^473^AKT), which was more evident at 48 hours of nitroxoline treatment (Fig. 2B). Consistently with the reduction of pSer^473^AKT, the expression of the Ser^9^ phosphorylated inactive form of GSK3β was drastically reduced, while the active unphosphorylated form was still expressed. Considering that the unphosphorylated form of GSK3β downregulates β-catenin, these findings are in line with the decreased levels of β-catenin observed in response to nitroxoline treatment (Fig. 2A).

### Nitroxoline impairs cell growth

**Figure 3:**
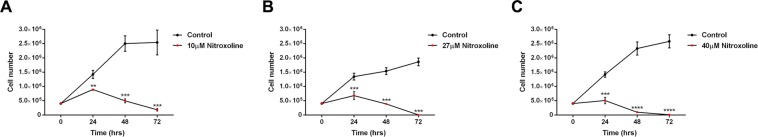
Nitroxoline affects cell growth. AsPC-1 cells were counted over a 72-hour time course treatment with vehicle (control), or nitroxoline at the indicated concentrations **(A–C)**. Data shown are the means ± SD of three determinations (**p < 0.01; ***p < 0.001; ****p < 0.0001).

Considering that ATP1B3 and PI3K/AKT/GSK3β/β-catenin pathway play an important role in cell growth, we verified whether nitroxoline treatment affected AsPC-1 growth (Fig. 3). In agreement with the downregulation of ATP1B3 and β-catenin, nitroxoline treatment was associated with a marked decrease in cell growth at all time points and drug concentrations analyzed. This effect of the drug was consistent with the antiproliferative effects that we had previously observed in pancreatic cancer cell lines treated with nitroxoline, which included decreased viability, impairment of cell cycle, apoptosis induction and reduction of clonogenic activity^7^.

### Nitroxoline impairs cell migration and invasion

**Figure 4:**
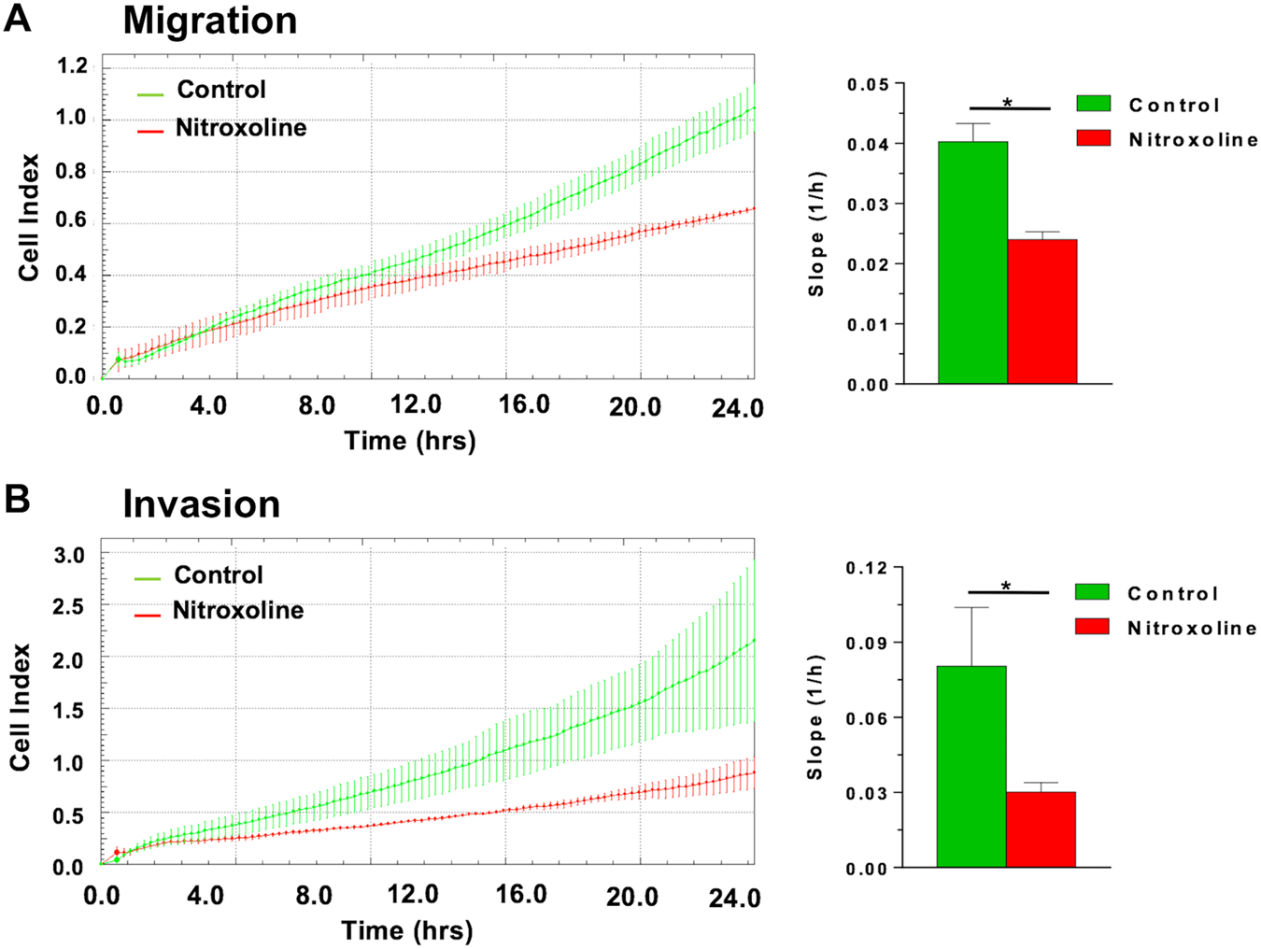
Nitroxoline impairs AsPC-1 motility. XCELLigence-driven dynamic monitoring of AsPC-1 migration (**A**) and invasion through diluted Matrigel **(B)** after 24-hours nitroxoline treatment. Each panel shows Cell Index curves (left) and slope analysis (right) that describe the steepness, incline, gradient, and changing rate of the Cell Index curves over time. All data are the mean of three separate experiments ± SD (*p < 0.05).

The PI3K/AKT/GSK3β/β-catenin pathway and ATP1B3 are known to play a crucial role in migration and invasion of different cancers^22,24,25^. Therefore, we assessed whether nitroxoline-induced downregulation of ATP1B3 and β-catenin was associated to alterations of AsPC-1 cell motility and/or invasion potential. In line with the effects of nitroxoline on ATP1B3 and β-catenin, treatment with this drug for 24 hours induced marked and significant decreases of both migration (Fig. 4A) and invasion (Fig. 4B), as determined by xCELLigence Real-Time Cell Analysis system.

### Nitroxoline increases intracellular ROS and activates oxidative DNA damage response

**Figure 5:**
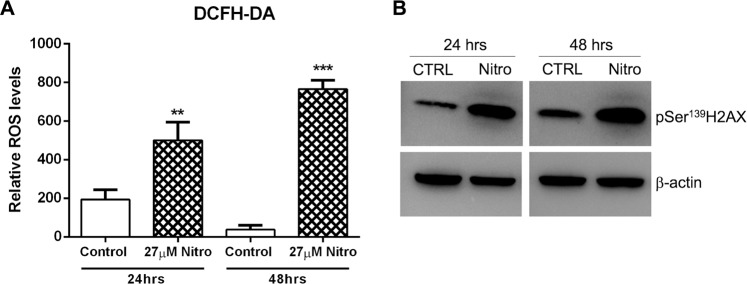
Nitroxoline promotes intracellular ROS production and Ser^139^H2AX phosphorylation. (**A**) Intracellular ROS levels in AsPC-1 cells were measured by flow cytometry using DCFH-DA after 24 and 48 hours of nitroxoline treatment. Data shown are the means ± SD of three independent experiments (**p < 0.01; ***p < 0.001). (**B**) Western blot analysis of pSer^139^H2AX at both 24 and 48 hours of nitroxoline treatment. Full-length of these representative western blots are showed in Supplementary Fig. S4.

Nitroxoline was previously shown to increase intracellular ROS levels in a lymphoma cell line^16^ and to induce DNA damage^17^. Notably, also Na/K-ATPase pump inhibition was previously shown to increase ROS production in different cell types^26–28^ and our proteomic analysis indicated that nitroxoline drastically decreased the expression of both the ATP1A1 and ATP1B3 subunits of the ion pump (Table 1). Therefore, we analyzed ROS production in response to nitroxoline treatment in AsPC-1 pancreatic cancer cells. Treatment with nitroxoline for 24 and 48 hours significantly induced a time-dependent increase of intracellular ROS production in this cell line (Fig. 5A). This finding shows that nitroxoline promotes oxidative stress, which may be at least in part related to the Na/K-ATPase pump downregulation indicated by proteomic analysis. Furthermore, considering that oxidative stress is known to induce phosphorylation of Histone Family Member X (H2AX) at Ser^139^ in response to DNA damage^29^, we tested whether nitroxoline treatment could associate with altered phosphorylation of this sensitive DNA damage marker. In line with increased ROS production, nitroxoline treatment induced a marked increase of pSer^139^H2AX at both 24- and 48-hours (Fig. 5B), suggesting that increased oxidative stress and DNA damage are among mechanisms through which this drug exerts its anticancer effect in AsPC-1 cells.

### Nitroxoline alters cell metabolism leading to mitochondrial depolarization

**Figure 6:**
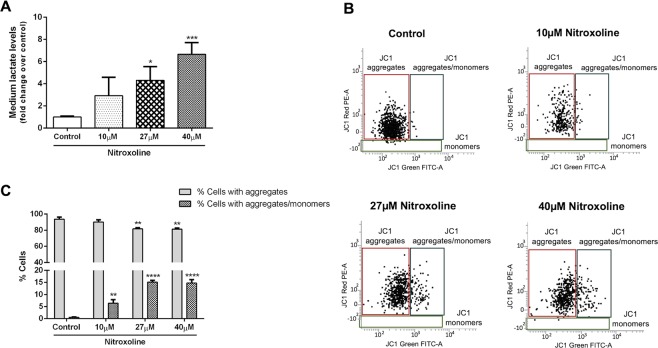
Nitroxoline promotes lactate production and mitochondrial membrane depolarization. AsPC-1 cells were treated for 48 hours with nitroxoline at the indicated concentrations, or with vehicle (control). (**A**) Histograms represent extracellular lactate levels in AsPC-1 cells culture media after treatment. Data shown are the means ± SD of three independent determinations (*p < 0.05; ***p < 0.001). (**B**) Cells were stained with the fluorescent probe JC-1 and analyzed by flow cytometry. Dot plots show representative experiments analyzing mitochondrial membrane potential in AsPC-1 cells after treatment. JC-1 aggregates emit red fluorescence, whereas JC-1 monomers emit green fluorescence. (**C**) Histograms show the mean percentage of cells with JC-1 aggregates or with aggregates and monomers. Data are the means ± SD of four experiments (**p < 0.01; ****p < 0.0001).

STRING analysis indicated that “TCA cycle and respiratory electron transport” and “Pyruvate metabolism and TCA cycle” pathways (REACTOME: HSA-1428517 and HSA-71406, respectively) were highly enriched (FDR: 4.70 × 10^−7^ and 3.02 × 10^−5^, respectively) among those consistently deregulated by nitroxoline (Supplementary Table 3). In particular, nitroxoline downregulated four TCA cycle enzymes (ACO2, CS, FH and SUCLA2), as well as four respiratory chain proteins (MT-CO2, NDUFS3, UQCRC1 and UQCRC2) and upregulated three glycolytic enzymes (ALDOA, LDHA and PGK1) (Table 1). Upregulation of ALDOA and PGK1 are expected to promote pyruvate metabolism, while downregulation of TCA cycle enzymes and upregulation of LDHA are expected to direct pyruvate toward lactate production. Furthermore, also enzymes of glutamine (GOT2, GLS, ALDH18A1) and lipid metabolism (ACAT1, ECHS1, HADHA, HADHB, PRDX6, FASN) resulted downregulated (Supplementary Table 2). The downregulation of these enzymes and the shift of pyruvate toward lactate predicted by the deregulation of glycolytic enzymes are expected to compromise fuel supply to TCA cycle, whose enzymes are also downregulated. Considering that TCA is a crucial convergence point in cellular respiration^30^, both reduced fuel supplies to the cycle and downregulation of its enzymes, together with the downregulation of respiratory chain proteins are expected to compromise mitochondrial potential, which is sustained by the proton gradient created by respiratory chain. In agreement with the upregulation of glycolytic enzymes and the downregulation of TCA cycle enzymes indicated by proteomic analysis, treatment of cells with nitroxoline induced a marked and significant increase of extracellular lactate levels in culture media (Fig. 6A). Moreover, in line with the deregulation of key metabolic enzymes and respiratory chain proteins, predicted to compromise mitochondrial potential, analysis of JC-1 (5,5′,6,6′-tetrachloro-1,1′,3,3′-tetraethyl-benzimidazolecarbocyanine iodide) by flow cytometry (Fig. 6B,C) showed that treatment with nitroxoline induced a significant decrease in the percentage of cells with functional mitochondria (displaying JC-1 aggregates). The drug induced also a marked increase in cells with depolarized mitochondria at all concentrations tested (displaying both JC-1 aggregates and monomers).

### Iron contributes to nitroxoline-induced cell death

**Figure 7:**
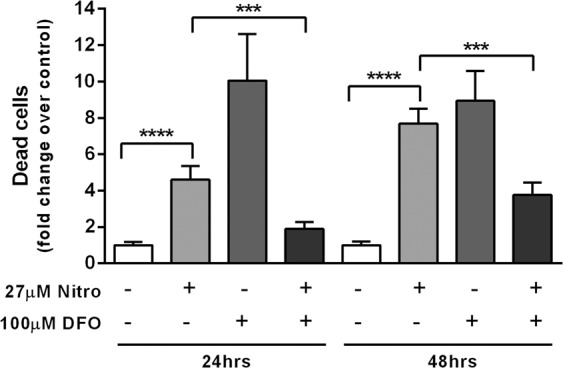
Iron chelator deferoxamine hampers nitroxoline-induced cell death. Changes in AsPC-1 cell death were measured by flow cytometry after 24 and 48 hours of treatment with vehicle (control), nitroxoline or deferoxamine as single agents, or in combination (***p < 0.001; ****p < 0.0001).

Oxidative stress is known to induce aconitase (ACO2) inactivation and subsequent TFRC protein overexpression, which in turn increases intracellular iron^31^, leading to apoptotic or non-apoptotic cell death^31,32^. Nitroxoline induced ROS production in AsPC-1 cells (Fig. 5A) and according to proteomic analysis ACO2 was the most downregulated protein in response to nitroxoline treatment, while TFRC was highly overexpressed at 24 and 48 hours after treatment with nitroxoline (Table 1 and Fig. 2A). Moreover, proteomic analysis showed that PCBP1 was highly downregulated in response to nitroxoline (Table 1). PCBP1 is a cytosolic iron chaperone involved in the delivery of iron to ferritin and its depletion in human cells is known to inhibit ferritin iron loading, leading to increased cytosolic iron pools^33^. Taken together, overexpression of TFRC and downregulation of PCBP1 suggested that increased cytosolic iron pools might play a role in nitroxoline-induced cell death. To explore this possibility, we analyzed whether the iron chelator deferoxamine modulated the ability of nitroxoline to induce AsPC-1 cell death (Fig. 7). Nitroxoline treatment at 24 and 48 hours induced a 5- to 8-fold increase in cell death with respect to vehicle control, as indicated by flow cytometry. In line with the crucial role of intracellular iron homeostasis in cancer cell biology^34^, also depletion of iron pools induced by deferoxamine as single agent caused a 9- to 10-fold increase in cell death. Notably, the combination of nitroxoline with deferoxamine drastically blunted cell death induced by either single agent at both 24 and 48 hours of treatment (Fig. 7). Taken together, the overexpression of TFRC and the downregulation of PCBP1 induced by nitroxoline, as well as the effect of the iron chelator deferoxamine are consistent with a role of iron in nitroxoline-induced cell death.

## Discussion

Pancreatic cancer is one of the most frequent causes of cancer death and its lethality is due in part to late diagnosis and to poor efficacy of current chemotherapeutic approaches^2^. Therefore, it is of paramount importance to develop more effective therapeutic approaches for the treatment of this lethal tumor. In this respect, repurposing of drugs in cancer may expand the repertoire of agents contributing to an improved treatment of PC.

In a recent study, we identified the antibacterial drug nitroxoline as a candidate for repurposing in pancreatic cancer, both as single agent and in combinations with other drugs^7^. At present, there is a poor understanding on how this antibacterial exerts its anticancer effects. A limited number of molecules and pathways that could be relevant in this regard were identified in studies designed to screen for specific target molecules or for molecular pathways known to play an important role in cancer biology^7,9,12–15,17–19^. Unbiased approaches analyzing the effects induced by nitroxoline in cancer cells, such as proteomic studies, have the potential to broaden our understanding of nitroxoline anticancer effects and to identify relevant proteins and pathways previously unknown to be affected by this drug. However, a proteomic analysis of nitroxoline effects in cancer cells has not been employed before.

In this study, we applied a shotgun proteomic approach to gain insights into the mechanisms of action of nitroxoline in AsPC-1 pancreatic cancer cells. By this unbiased approach we identified a total of 363 proteins deregulated by nitroxoline, which included 81 proteins consistently affected by nitroxoline treatment at both 24- and 48-hours. Most of these proteins had not been previously linked to the effect of nitroxoline in cancer cells. Notably, STRING analysis showed that the majority of the 81 proteins were connected within a single network, with a highly significant protein-protein interaction enrichment score, suggesting that the bulk of nitroxoline effects can be ascribed to up-regulated or down-regulated expression of proteins interacting within this network. In line with this possibility, STRING analysis predicted that also molecules previously known to be affected by this drug interact with proteins included in the main functional interaction network.

IPA evaluation of our proteomic results supported an important role for some proteins deregulated by nitroxoline in previous studies^12,15,17^. In particular, proteomic analysis indicated that sirtuin and mTOR signaling, known to be modulated by nitroxoline^15,17^, were among the most highly enriched pathways consistently deregulated by the drug. Moreover, the PI3K/AKT pathway that was recently shown to be downregulated by nitroxoline in prostate cancer cells^12^ emerged as one of the Canonical Pathways highly enriched by IPA in our proteomic analysis. Intriguingly, sirtuin and mTOR were previously reported to be upregulated in gemcitabine-resistant PC cells^35^. In addition, inhibition of PI3K, an enzyme downregulated by nitroxoline in our study, was previously shown to sensitize to gemcitabine^36^. Similarly, survivin and Bcl-2 proteins were reported to be upregulated in gemcitabine-resistant PC cells, whereas their expression was decreased by nitroxoline in several studies^12,13,19,35^. Taken together, these observations indicate that nitroxoline modulates molecules that have a key role in gemcitabine resistance, suggesting that it may affect sensitivity to this chemotherapeutic agent. Further studies will be necessary to investigate this hypothesis.

Notably, IPA and STRING analyses of proteomic results identified also many highly enriched, consistently deregulated pathways previously unknown to be modulated by the drug, such as those related to metabolism, to protein translation and to co-translational membrane targeting. Considering that downregulation of proteins involved in translation and targeting to membrane was paralleled by a reduction of cell surface protein labeling by flow cytometry and cancer cells depend on efficient protein translation and translocation into the endoplasmic reticulum to support their fast growth^37^, impairment of these processes is likely to contribute to nitroxoline antiproliferative effects. In this regard, inhibition of translocation is considered an interesting target for the development of new anticancer drugs^37^.

Among the key oncogenic proteins previously unknown to be downregulated by nitroxoline, β-catenin is known to play a crucial role in cell proliferation, survival and stemness, contributing to carcinogenesis and tumor progression of pancreatic cancer and other tumors^38^. Downregulation of this molecule is a strategy that is currently being considered in cancer treatment^38^. In the present study, downregulation of β-catenin in response to nitroxoline treatment was associated with a marked and time-dependent decrease in cell growth. In line with the reduced cell growth observed in this study, we have previously shown that nitroxoline treatment decreased viability, affected cell cycle, reduced the expression of relevant cell cycle proteins, induced apoptosis and drastically impaired clonogenic activity in pancreatic cancer cell lines^7^. All of these effects are consistent with the downregulation of β-catenin induced by nitroxoline^39^. Moreover, also the reduction in migration and invasion observed in the present study in response to nitroxoline treatment are consistent with the downregulation of β-catenin^40^.

The ATP1B3 and ATP1A1 subunits of Na/K-ATPase pump were among additional proteins consistently downregulated by nitroxoline in our proteomic analysis. Several effects of the drug in AsPC-1 appear to be related to downregulation of this pump that had not been previously implicated in nitroxoline action. The Na/K-ATPase pump plays a crucial role in tumor cells and is currently considered a potential target for the development of anti-cancer drugs^22,41^. It was previously shown that knockdown of the Na ^+^/K ^+^ -ATPase α1 subunit impairs migration and proliferation of lung cancer cells^42^. Na ^+^/K ^+^ -ATPase inhibitors induce cell cycle arrest and apoptosis in several human cancer cells, such as those derived from hepatoma^43^, lung^44^ and pancreatic cancer^45^. Inhibition of Na ^+^/K^+^-ATPase pump was shown to impair the key oncogenic PI3K/AKT signalling pathway in gastric cancer cells^46^. Inhibiting of Na ^+^/K^+^-ATPase pump suppresses hepatoma cell adhesion, migration, and invasion through inhibition of PI3K/AKT/mTOR pathway^41,47^. In gastric cancer cells knockdown of ATP1B3 expression inhibited proliferation, colony-formation ability, migration, and invasion and induced apoptosis via the PI3K/AKT pathway^22^. Therefore, the downregulation of Na ^+^/K^+^-ATPase subunits in response to nitroxoline treatment, revealed by our proteomic analysis, had the potential to play a key role in several of the antitumor effects of this drug. Consistently with the link between the Na ^+^/K^+^-ATPase pump and the PI3K/AKT pathway previously described in several cancer cell lines^22,45–47^, also in our study we observed that the decreased expression of ATP1B3 and ATP1A1 subunits was associated with decreased expression of PI3K, pSer^473^AKT, pSer^9^GSK3β and with downregulation of β-catenin. In view of their crucial role in cancer cell viability, it is conceivable that downregulation of Na ^+^/K^+^-ATPase subunits and hampering of PI3K/AKT/GSK3β/β-catenin pathway contribute to cell growth inhibition, viability and clonogenicity impairment, as well as cell death induction in pancreatic cancer cells treated with nitroxoline, which we observed both in the present and in a previous study^7^. In addition, considering the involvement of Na ^+^/K ^+^ -ATPase pump and PI3K/AKT pathway in cell migration and invasion^22,24,41^, it is likely that their downregulation induced by nitroxoline plays a role in the impairment of AsPC-1 migration and invasion. Thus, the decreased expression of Na ^+^/K^+^-ATPase subunits, associated with the downregulation of the PI3K/AKT/GSK3β/β-catenin pathway, emerges as a mechanism by which nitroxoline induces antitumor effects in AsPC-1 pancreatic cancer cells.

Nitroxoline had been previously shown to increase ROS production in lymphoma and ovarian cancer cells lines^16^. We observed that nitroxoline increased ROS levels in AsPC-1 cells. Intriguingly, both downregulation and inhibition of the Na ^+^/K^+^-ATPase pump were previously shown to increase ROS production in cancer cells^41,48^, suggesting a link between nitroxoline-induced downregulation of the pump and increased ROS production observed in the present study. Excessive ROS production is known to induce DNA damage, which in turn activates cell-cycle checkpoints, leading to cell-cycle arrest and prevention of damaged DNA replication^49^. Induction of DNA damage by nitroxoline had been previously described in prostate cancer cells^17^. In line with these previous observations, we found that nitroxoline-induced downregulation of Na^+^/K^+^-ATPase subunits was paralleled by a drastic and time-dependent increase in phosphorylation of H2AX at Ser^139^, a sensitive marker of DNA damage response. Increased ROS production and activation of DNA damage response explain at least in part the effects of nitroxoline on cell cycle, including the downregulation of cyclin B1 and cyclin D3, which we had previously described in PC cells^7^. Overall, also nitroxoline effects on cell cycle might be related to Na/K-ATPase pump downregulation.

The previously unknown deregulation induced by nitroxoline on several pathways related to metabolism, leading to compromised mitochondrial potential, was a major finding of this study. In particular, nitroxoline downregulated enzymes of TCA cycle, which is emerging as an attractive therapeutic target in cancer^30^. Furthermore, the downregulation of TCA cycle enzymes and respiratory electron transfer proteins, together with the concomitant deregulation of metabolic enzymes necessary for fuel supply to TCA cycle in response to nitroxoline treatment, were associated with a marked increase in the percentage of cells with depolarized mitochondria. Deregulation of metabolic enzymes, together with downregulation of respiratory chain molecules and the resulting mitochondrial depolarization, provide a new mechanism for nitroxoline anticancer effects. In particular, this mechanism may contribute to nitroxoline-induced apoptosis, which we have previously described to occur in pancreatic cancer cells treated with the drug^7^.

Nitroxoline treatment increased expression of TFRC and downregulation of PCBP1 and these alterations were previously shown to increase cytosolic iron pools^31,33^. Regulation of cytosolic iron pools appears to be critical in cancer biology. Increased iron levels and increased expression of TFRC may either contribute to tumorigenesis and cancer growth^50^, or be detrimental for cancer cell viability, leading to apoptotic and non-apoptotic cell death^31,32^. Furthermore, even the depletion of intracellular iron by deferoxamine may reduce growth and induce apoptosis in cancer cell lines^34^. In line with the critical regulation of iron pool homeostasis in cancer biology, in our study both iron chelation by deferoxamine, as well as upregulation of TFRC and downregulation of PCBP1 induced by nitroxoline, were associated with increased cell death, whereas the combination of deferoxamine and nitroxoline drastically blunted the effect of either agent on cell death. Therefore, taken together, these observations suggest that altered iron metabolism might contribute to the anticancer effects of nitroxoline and further studies will be necessary to assess this possibility.

In summary, this is the first study analyzing the effect of the repurposed drug candidate nitroxoline on the proteome of cancer cells. Unbiased proteomic analysis was integrated by western blot and by functional assays that evaluated processes playing a crucial role in cancer biology, such as cell growth, migration, invasion, ROS generation, DNA damage, protein translocation, mitochondrial membrane polarization and cell death. The results of these integrative analyses provide novel insights into the mechanisms of nitroxoline antitumor action in AsPC-1 pancreatic cancer cells. The drug modulates multiple biological pathways and key oncogenic proteins previously unknown to play a role in nitroxoline anticancer effect. Bioinformatic analyses showed also that the network of proteins and pathways deregulated by the drug in our study is connected to proteins deregulated by nitroxoline in previous studies based on candidate target approaches. In conclusion, the present study broadens our understanding of the pleiotropic mechanism of nitroxoline anticancer action, providing a model that could be applied to the study of other repurposed drug candidates.

## Materials and Methods

### Proteomic studies

The human PC cell line AsPC-1 was purchased from Cell Lines Service (CLS, Eppelheim, Germany) and cultured as described^7^. Nitroxoline was purchased from Sigma (St. Louis, MO, USA). Cells were seeded in 100 mm culture dishes (2 × 10^6^ cells/dish) and the following day, cells were treated with 27 µM nitroxoline, corresponding to the IC50 value for this drug in AsPC-1 cells^7^, or with vehicle (Dimethyl Sulfoxide, DMSO) for 24 or 48 hours. Then, cells were dissociated with trypsin-EDTA 1X in Phosphate-Buffered Saline (PBS) and pelleted.

#### Protein extraction and Filter-aided sample preparation

Pelleted cells from three independent replicates for each timepoint were lysed by adding 200 μL of buffer containing urea 8 M, Tris 10 mM, dithiothreitol 50 mM, SDS 2% and Triton X-100 0.4% and centrifuged at 13,000 × g for 15 min to remove the insoluble fraction. Proteins in the supernatant were quantified through the Bradford assay. A volume corresponding to 50 μg of proteins was loaded onto a Nanosep 10-kDa-cutoff filter (Pall Corporation – Michigan USA) and digested according to a previously described protocol adapted from Distler^51^. Briefly the sample was washed twice with 200 μL urea buffer (8 M urea, 100 mM Tris pH 8.5 in Milli-Q water) to remove detergents present in the lysis buffer. Proteins on the filter were subsequently reduced and alkylated by adding 100 μL of DTT solution (8 mM dithiothreitol in urea buffer) and 100 μL of IAA solution (50 mM iodoacetamide in urea buffer). The buffer was exchanged with 50 mM ammonium bicarbonate (pH 7.8) before adding the trypsin solution (Sigma, St. Louis, MO, USA) for protein digestion to a ratio of 1:50. The reaction was incubated for 16 hours at 37 °C, and the mixture of peptides was collected by centrifugation, acidified with 10% trifluoroacetic acid (Sigma, St. Louis, MO, USA) and stored at −20 °C until analysis.

#### LC-MS/MS label free shotgun proteomics

Digested proteins were analysed in technical triplicates by LC-MS/MS using a Proxeon EASY-nLCII (Thermo Fisher Scientific, Milan, Italy) chromatographic system coupled to a Maxis HD UHR-TOF (Bruker Daltonics GmbH, Bremen, Germany) mass spectrometer. Peptides were loaded on the EASY-Column C18 trapping column (2 cm L., 100 µm I.D, 5 µm ps, Thermo Fisher Scientific), and subsequently separated on an Acclaim PepMap100 C18 (75 µm I.D., 25 cm L, 5 µm ps, Thermo Fisher Scientific) nano scale chromatographic column. The flow rate was set to 300 nL/min and the gradient was from 3 to 35% of B in 80 min followed by 35 to 45% in 10 min and from 45 to 90% in 11 min. Mobile phase A was 0.1% formic acid in H_2_O and mobile phase B was 0.1% formic acid in acetonitrile. The mass spectrometer was equipped with a nanoESI spray source and operated in positive ion polarity and Auto MS/MS mode (Data Dependent Acquisition - DDA). Precursors in the range 350 to 2,200 m/z (excluding 1,220.0–1,224.5 m/z) with a preferred charge state +2 to +5 (excluding singly charged ions). After acquiring one MS/MS spectrum, the precursors were actively excluded from selection for 30 seconds. In-source reference lock mass (1,221.9906 m/z) was acquired online throughout the runs.

#### Bioinformatics processing

Raw data were processed using PEAKS Studio v7.5 software (Bioinformatic Solutions Inc, Waterloo, Canada) using the ‘correct precursor only’ option. The resulting mass lists were searched against nextprot database (including isoforms as of June 2017; 42,151 entries). Carbamidomethylation of cysteines was selected as fixed modification, oxidation of methionines and deamidation of asparagine and glutamine were set as variable modifications. Non-specific cleavage was allowed to one end of the peptides, with a maximum of 2 missed cleavages. The highest error mass tolerances for precursors and fragments were set at 10 ppm and 0.05 Da, respectively. Quantitative information was obtained by label free quantification analysis performed using the integrated tool PEAKS-Q, part of the PEAKS Studio suite.

Gene Ontology and pathway analysis were performed by Ingenuity Pathway Analysis (IPA, QIAGEN Redwood City, www.qiagen.com/ingenuity, Build version: 321501 M, Content version: 21249400). The analysis was performed using the core analysis option and considering only direct relationships among genes. Protein-protein interaction networks and functional enrichment were evaluated by STRING (v. 11.0) analysis^52^.

### Western blot analysis

Immunoblotting was performed essentially as described^7^, except that the Westar ηC Ultra 2.0 chemiluminescence substrate (Cyanagen, Bologna, Italy) was employed. Rabbit monoclonal anti-ATP1B3 and rabbit polyclonal anti-transferrin receptor antibodies were purchased from Abcam (Cambridge, England). Mouse monoclonal anti β-catenin antibody was obtained from Santa Cruz Biotechnology, Inc. (Dallas, TX, USA). Rabbit monoclonal PI3 Kinase p100α, rabbit polyclonal AKT, rabbit polyclonal phospho-AKT (Ser^473^), rabbit monoclonal GSK3β, rabbit polyclonal phospho-GSK3β (Ser^9^) and phospho-Histone H2A.X (Ser^139^) antibodies were obtained from Cell Signaling Technology, Inc. (Beverly, MA, USA). Monoclonal anti-β-actin antibody was purchased from Sigma (St. Louis, MO, USA).

### Cell growth assay

AsPC-1 cells were seeded in 6-well plates (4 × 10^5^ cells/well) and were treated with vehicle (DMSO) or nitroxoline at the indicated concentrations. Cells were counted at 24, 48 and 72 hours using the trypan blue exclusion test.

### Real-Time assays of cell migration and invasion

Cells were treated for 24 hours with 27 µM nitroxoline and then subjected to migration and invasion assays by means of the xCELLigence RTCA system (Real-Time Cell Analyzer System, Acea Biosciences Inc., San Diego, CA) essentially as previously reported^53^. The system was developed to monitor cell events in real time, without incorporation of dyes, by measuring electrical impedance. For migration assays, 4 × 10^5^ cells/well were seeded onto the top chambers of CIM-16 plates and the bottom chambers were filled with medium containing 5% fetal bovine serum (FBS) as chemoattractant. For analysis of invasiveness, 4 × 10^5^ cells/well were seeded onto the upper chambers covered with a layer of diluted Matrigel (BD Biosciences) and the bottom chambers were filled with medium containing 10% FBS. For both migration and invasion assays, each condition was performed in triplicate and the signal detection was programmed every 15 min for a total of 24 hours. Impedance values were expressed as a dimensionless parameter termed Cell Index (CI). The rate of cell migration and invasion was also determined by calculating the slope, that describes the steepness, incline, gradient and changing rate of the Cell Index curves over time.

### Flow cytometry analyses

#### Detection of reactive oxygen species (ROS)

ROS production was measured using 2′,7′-dichlorofluorescin diacetate (DCFH-DA, Sigma, St. Louis, MO, USA) by monitoring the increase of the related green fluorescence. Fluorescent emission was analyzed by a FACScanto II flow cytometer (BD, Becton-Dickinson Biosciences, San Jose, CA) after labeling cells with 10 µM of DCFH-DA for 45 min at 37 °C, in the dark. For each sample, 10,000 events were recorded. To evaluate non-specific fluorescence, corresponding unstained samples were acquired and DCFH-DA mean fluorescence intensity (MFI) ratio values were standardized by dividing the MFI of positive events by the MFI of negative events^54^.

#### Analysis of mitochondrial activity

Mitochondrial membrane potentials were measured using the fluorescent probe JC-1 (Sigma, St. Louis, MO, USA). Cells were stained with JC-1 fluorescent probe (1 µM) for 45 min at 37 °C in the dark. JC-1 forms aggregates that emit red fluorescence at the high concentrations reached in energized mitochondria. These aggregates are spectrally distinguishable from dye monomers formed in de-energised or depolarized mitochondria that emit green fluorescence. For each sample, 10,000 events were recorded. Fluorescent emission was analyzed, in terms of MFI ratio values, as described above. Populations of cells with energized or depolarized mitochondria were separated by gating in two-parameter measurement space^55^.

#### Iron chelation and cell viability

The effect of iron chelation by deferoxamine (Sigma, St. Louis, MO, USA) on cell viability, with or without nitroxoline treatment, was determined using 7-Amino Actinomycin D (7-AAD, Via-Probe, BD Biosciences, Cat. 555815). For each sample, 10,000 events were recorded.

#### Flow cytometry acquisition and analysis

Samples were acquired by using a FACSVerse flow cytometer (BD Biosciences - three laser, eight colour configuration). Data reproducibility and flow cytometer performances were monitored by the Cytometer Setup & Tracking Module (BD Biosciences). Compensation were assessed using CompBeads (BD) and single stained fluorescent samples. Data were analyzed using FACSDiva v 6.1.3 (BD), FACSuite v 1.0.5 (BD) and FlowJo v 8.8.6 (TreeStar, Ashland, OR, USA) software.

### Lactate measurement in cell culture medium

Lactate levels in growth media of AsPC-1 cells were measured as previously described^56^. Briefly, cells were seeded in 6-well plates (3.5 × 10^5^ cells/well) and treated for 48 hours with vehicle (DMSO), or nitroxoline at the indicated concentrations. Then, culture supernatants were analyzed for lactate levels using Lactate Pro Analyser (Arkray Inc. Kyoto, Japan).

### Statistical analysis

Statistical analyses were performed using GraphPad Prism version 5.01 software (San Diego, CA). Comparisons of mean values were performed by an unpaired Student’s t-test. A p-value ≤ 0.05 was considered statistically significant.

## Supplementary information

Supplementary Table 1.

Supplementary Information.

Supplementary Table 4.

## Data Availability

All data underlying the findings described within the manuscript are fully available without restrictions.
