# Supplementary material for: Integrative proteomic and functional analyses provide novel insights into the action of the repurposed drug candidate nitroxoline in AsPC-1 cells
Source: Sci Rep. 2020 Feb 13;10:2574. doi: 10.1038/s41598-020-59492-4 (PMC7018951; doi:10.1038/s41598-020-59492-4)
Supplement: Supplementary file 2 — Supplementary Information. [file 41598_2020_59492_MOESM2_ESM.pdf]

Supplementary Information.

Integrative proteomic and functional analyses provide novel insights into the action of the repurposed drug candidate nitroxoline in AsPC-1 cells

Serena Veschi[1#], Maurizio Ronci[2,3#], Paola Lanuti[3,4], Laura De Lellis[1], Rosalba Florio[1], Giuseppina Bologna[3,4], Luca Scotti[2], Erminia Carletti[2,3], Federica Brugnoli[5], Maria Cristina Di Bella[1], Valeria Bertagnolo[5], Marco Marchisio[3,4], Alessandro Cama[1,3*]

[1]Department of Pharmacy, G. d'Annunzio University of Chieti-Pescara, Chieti, Italy; [2]Department of Medical, Oral and Biotechnological Sciences, G. d'Annunzio University of Chieti-Pescara, Chieti, Italy; [3]Centre on Aging Sciences and Translational Medicine (Ce.S.I-Me.T), G. d'Annunzio University of Chieti-Pescara, Chieti, Italy; [4]Department of Medicine and Aging Sciences, G. d'Annunzio University of Chieti-Pescara, Chieti, Italy; [5]Section of Anatomy and Histology, Department of Morphology, Surgery and Experimental Medicine, University of Ferrara, Ferrara, Italy.

# These authors contributed equally to this work

***Correspondence to**: Alessandro Cama, MD, Department of Pharmacy, G. d'Annunzio University of Chieti-Pescara, Via dei Vestini, 66100, Chieti, Italy; e-mail: alessandro.cama@unich.it; tel: +39-0871-3554559; fax: +39-0871-3554557

INDEX PAGE

Supplementary Table 2 S3

Supplementary Table 3 S4

Supplementary Figure S1 S5

Supplementary Figure S2 S6

Supplementary Figure S3 (full-length blots of Figure 2, panel A) S7

Supplementary Figure S4 (full-length blots of Figure 2, panel B and of Figure 5, panel B) S8

Supplementary Table 2. Proteins consistently deregulated at 24 and 48 hours of treatment with nitroxoline, resulted from the intersection of the lists in Supplementary Table 1 (190 and 254 hits respectively as shown in the Venn diagram).

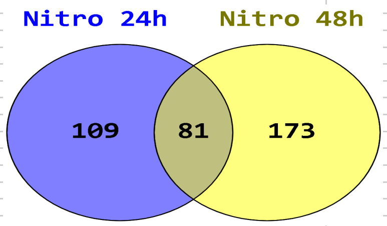

Description	Accession	Gene	Ratio nitroxoline *vs* vehicle control 24h	48h
Plasminogen activator inhibitor 2 (PAI-2)	P05120	SERPINB2	5,76	100,00
Prolyl 4-hydroxylase subunit alpha-1 (4-PH alpha-1)	P13674	P4HA1	33,96	100,00
Laminin subunit alpha-3 (Epiligrin 170 kDa subunit) (E170)	Q16787	LAMA3	100,00	100,00
Tumor-associated calcium signal transducer 2 (Cell surface glycoprotein Trop-2)	P09758	TACSTD2	8,46	12,50
ERO1-like protein alpha (ERO1-L) (ERO1-L-alpha)	Q96HE7	ERO1A	2,72	10,00
Transferrin receptor protein 1	P02786	TFRC	3,74	5,88
Phosphoglycerate kinase 1	P00558	PGK1	2,47	5,26
Solute carrier family 2, facilitated glucose transporter member 3	P11169	SLC2A3	3,30	5,26
Laminin subunit beta-3 (Epiligrin subunit bata)	Q13751	LAMB3	100,00	5,00
Ephrin type-A receptor 2	P29317	EPHA2	2,18	4,76
Integrin alpha-2 (CD49 antigen-like family member B)	P17301	ITGA2	2,05	4,55
Kunitz-type protease inhibitor 2	O43291	SPINT2	2,45	3,33
Aspartyl/asparaginyl beta-hydroxylase	Q12797	ASPH	0,47	3,03
14-3-3 protein sigma (Epithelial cell marker protein 1)	P31947	SFN	2,07	2,94
Fructose-bisphosphate aldolase A	P04075	ALDOA	2,57	2,32
L-lactate dehydrogenase A chain (LDH-A)	P00338	LDHA	2,39	2,17
Epiplakin (450 kDa epidermal antigen)	P58107	EPPK1	4,94	2,00
Citrate synthase, mitochondrial	O75390	CS	0,49	0,50
Adenosylhomocysteinase (AdoHcyase)	P23526	AHCY	0,44	0,49
Histone H2A type 2-B	Q8IUE6	HIST2H2AB	0,29	0,48
60S ribosomal protein L37a (Large ribosomal subunit protein eL43)	P61513	RPL37A	0,45	0,48
60S ribosomal protein L31 (Large ribosomal subunit protein eL31)	P62899	RPL31	0,28	0,47
40S ribosomal protein S5 (Small ribosomal subunit protein uS7)	P46782	RPS5	0,38	0,46
Eukaryotic initiation factor 4A-I (eIF-4A-I) (eIF4A-I)	P60842	EIF4A1	0,40	0,43
Non-histone chromosomal protein HMG-17	P05204	HMGN2	0,34	0,43
60S ribosomal protein L19 (Large ribosomal subunit protein eL19)	P84098	RPL19	0,30	0,42
40S ribosomal protein S10 (Small ribosomal subunit protein eS10)	P46783	RPS10	0,32	0,41
Dynamin-like 120 kDa protein, mitochondrial	O60313	OPA1	0,46	0,41
60S ribosomal protein L28 (Large ribosomal subunit protein eL28)	P46779	RPL28	0,31	0,40
40S ribosomal protein S26 (Small ribosomal subunit protein eS26)	P62854	RPS26	0,36	0,39
Heterogeneous nuclear ribonucleoprotein A0 (hnRNP A0)	Q13151	HNRNPA0	0,41	0,39
60S ribosomal protein L29 (Cell surface heparin-binding protein HIP)	P47914	RPL29	0,41	0,39
Fumarate hydratase, mitochondrial (Fumarase) (EC 4.2.1.2)	P07954	FH	0,29	0,38
5'-3' exoribonuclease 2	Q9H0D6	XRN2	0,47	0,38
Protein SET (HLA-DR-associated protein II)	Q01105	SET	0,43	0,37
Nascent polypeptide-associated complex subunit alpha, muscle-specific form	E9PAV3	NACA	0,39	0,36
Isoleucine--tRNA ligase, mitochondrial (EC 6.1.1.5) (Isoleucyl-tRNA synthetase) (IleRS)	Q9NSE4	IARS2	0,00	0,35
Poly(rC)-binding protein 1 (Alpha-CP1)	Q15365	PCBP1	0,50	0,35
Cytochrome b-c1 complex subunit 1, mitochondrial (Complex III subunit 1)	P31930	UQCRC1	0,42	0,34
T-complex protein 1 subunit beta (TCP-1-beta) (CCT-beta)	P78371	CCT2	0,41	0,34
60S ribosomal protein L27 (Large ribosomal subunit protein eL27)	P61353	RPL27	0,49	0,33
Elongation factor Tu, mitochondrial (EF-Tu) (P43)	P49411	TUFM	0,41	0,33
Catenin beta-1 (Beta-catenin)	P35222	CTNNB1	0,35	0,32
Complement component 1 Q subcomponent-binding protein, mitochondrial	Q07021	C1QBP	0,34	0,31
Sodium/potassium-transporting ATPase subunit alpha-1 (Na(+)/K(+) ATPase alpha-1 subunit	P05023	ATP1A1	0,49	0,30
ADP/ATP translocase 3 (ADP,ATP carrier protein 3)	P12236	SLC25A6	0,30	0,28
Protein arginine N-methyltransferase 1	Q99873	PRMT1	0,33	0,27
10 kDa heat shock protein, mitochondrial (Hsp10) (10 kDa chaperonin)	P61604	HSPE1	0,47	0,27
Aspartate aminotransferase, mitochondrial (mAspAT)	P00505	GOT2	0,35	0,26
Nucleoside diphosphate kinase B (NDK B) (NDP kinase B)	P22392	NME2	0,37	0,22
Cytochrome c oxidase subunit 2 (Cytochrome c oxidase polypeptide II)	P00403	MT-CO2	0,42	0,22
Trifunctional enzyme subunit alpha, mitochondrial (78 kDa gastrin-binding protein)	P40939	HADHA	0,32	0,22
T-complex protein 1 subunit epsilon (TCP-1-epsilon) (CCT-epsilon)	P48643	CCT5	0,41	0,19
T-complex protein 1 subunit zeta (TCP-1-zeta)	P40227	CCT6A	0,23	0,19
Enoyl-CoA hydratase, mitochondrial	P30084	ECHS1	0,45	0,19
40S ribosomal protein S17 (Small ribosomal subunit protein eS17)	P08708	RPS17	0,49	0,19
Cytochrome b-c1 complex subunit 2, mitochondrial (Complex III subunit 2)	P22695	UQCRC2	0,36	0,19
40S ribosomal protein S7 (Small ribosomal subunit protein eS7)	P62081	RPS7	0,50	0,18
ATPase family AAA domain-containing protein 3A	Q9NVI7	ATAD3A	0,48	0,18
Proliferation-associated protein 2G4 (Cell cycle protein p38-2G4 homolog)	Q9UQ80	PA2G4	0,45	0,18
Signal recognition particle 14 kDa protein (SRP14) (18 kDa Alu RNA-binding protein)	P37108	SRP14	0,30	0,16
60S ribosomal protein L18a (Large ribosomal subunit protein eL20)	Q02543	RPL18A	0,48	0,15
Peroxiredoxin-6	P30041	PRDX6	0,38	0,15
Profilin-1 (Epididymis tissue protein Li 184a)	P07737	PFN1	0,47	0,11
Glutaminase kidney isoform, mitochondrial (GLS)	O94925	GLS	0,15	0,11
Stathmin (Leukemia-associated phosphoprotein p18) (Metablastin) (Oncoprotein 18)	P16949	STMN1	0,45	0,11
Succinate--CoA ligase [ADP-forming] subunit beta, mitochondrial	Q9P2R7	SUCLA2	0,40	0,11
Trifunctional enzyme subunit beta, mitochondrial	P55084	HADHB	0,44	0,10
Acetyl-CoA acetyltransferase, mitochondrial	P24752	ACAT1	0,42	0,10
NADPH:adrenodoxin oxidoreductase, mitochondrial (AR) (Adrenodoxin reductase)	P22570	FDXR	0,01	0,09
NADH dehydrogenase [ubiquinone] iron-sulfur protein 3, mitochondrial	O75489	NDUFS3	0,28	0,08
Fatty acid synthase	P49327	FASN	0,10	0,08
40S ribosomal protein S23 (Small ribosomal subunit protein uS12)	P62266	RPS23	0,46	0,08
Sodium/potassium-transporting ATPase subunit beta-3	P54709	ATP1B3	0,38	0,08
40S ribosomal protein S24 (Small ribosomal subunit protein eS24)	P62847	RPS24	0,34	0,05
Eukaryotic translation initiation factor 4B (eIF-4B)	P23588	EIF4B	0,13	0,00
Delta-1-pyrroline-5-carboxylate synthase	P54886	ALDH18A1	0,40	0,00
60S ribosomal protein L38 (Large ribosomal subunit protein eL38)	P63173	RPL38	0,50	0,00
Urokinase plasminogen activator surface receptor	Q03405	PLAUR	14,18	0,00
G-rich sequence factor 1 (GRSF-1)	Q12849	GRSF1	0,00	0,00
Aconitate hydratase, mitochondrial (Aconitase)	Q99798	ACO2	0,00	0,00

Supplementary Table 3. Most significant enriched REACTOME pathways and functionally related protein groups obtained by STRING analysis of the 81 proteins consistently modulated at 24 and 48 hours by nitroxoline.

ID	Pathway	proteins	false discovery rate
HSA-156827	L13a-mediated translational silencing of Ceruloplasmin expression	EIF4A1,EIF4B,RPL18A,RPL19,RPL27,RPL28,RPL29,RPL31,RPL37A,RPL38,RPS10,RPS17,RPS23,RPS24,RPS26,RPS5,RPS7	3.38e-19
HSA-192823	Viral mRNA Translation	GRSF1,RPL18A,RPL19,RPL27,RPL28,RPL29,RPL31,RPL37A,RPL38,RPS10,RPS17,RPS23,RPS24,RPS26,RPS5,RPS7	3.38e-19
HSA-72706	GTP hydrolysis and joining of the 60S ribosomal subunit	EIF4A1,EIF4B,RPL18A,RPL19,RPL27,RPL28,RPL29,RPL31,RPL37A,RPL38,RPS10,RPS17,RPS23,RPS24,RPS26,RPS5,RPS7	3.38e-19
HSA-1799339	SRP-dependent cotranslational protein targeting to membrane	RPL18A,RPL19,RPL27,RPL28,RPL29,RPL31,RPL37A,RPL38,RPS10,RPS17,RPS23,RPS24,RPS26,RPS5,RPS7,SRP14	3.04e-18
HSA-156902	Peptide chain elongation	RPL18A,RPL19,RPL27,RPL28,RPL29,RPL31,RPL37A,RPL38,RPS10,RPS17,RPS23,RPS24,RPS26,RPS5,RPS7	3.87e-18
HSA-2408522	Selenoamino acid metabolism	AHCY,RPL18A,RPL19,RPL27,RPL28,RPL29,RPL31,RPL37A,RPL38,RPS10,RPS17,RPS23,RPS24,RPS26,RPS5,RPS7	3.87e-18
HSA-168254	Influenza Infection	GRSF1,RPL18A,RPL19,RPL27,RPL28,RPL29,RPL31,RPL37A,RPL38,RPS10,RPS17,RPS23,RPS24,RPS26,RPS5,RPS7,SLC25A6	5.70e-18
HSA-2408557	Selenocysteine synthesis	RPL18A,RPL19,RPL27,RPL28,RPL29,RPL31,RPL37A,RPL38,RPS10,RPS17,RPS23,RPS24,RPS26,RPS5,RPS7	5.70e-18
HSA-72764	Eukaryotic Translation Termination	RPL18A,RPL19,RPL27,RPL28,RPL29,RPL31,RPL37A,RPL38,RPS10,RPS17,RPS23,RPS24,RPS26,RPS5,RPS7	5.70e-18
HSA-975956	Nonsense Mediated Decay (NMD) independent of the Exon Junction Complex (EJC)	RPL18A,RPL19,RPL27,RPL28,RPL29,RPL31,RPL37A,RPL38,RPS10,RPS17,RPS23,RPS24,RPS26,RPS5,RPS7	5.70e-18
HSA-72689	Formation of a pool of free 40S subunits	RPL18A,RPL19,RPL27,RPL28,RPL29,RPL31,RPL37A,RPL38,RPS10,RPS17,RPS23,RPS24,RPS26,RPS5,RPS7	1.16e-17
HSA-72766	Translation	EIF4A1,EIF4B,IARS2,RPL18A,RPL19,RPL27,RPL28,RPL29,RPL31,RPL37A,RPL38,RPS10,RPS17,RPS23,RPS24,RPS26,RPS5,RPS7,SRP14,TUFM	1.54e-17
HSA-975957	Nonsense Mediated Decay (NMD) enhanced by the Exon Junction Complex (EJC)	RPL18A,RPL19,RPL27,RPL28,RPL29,RPL31,RPL37A,RPL38,RPS10,RPS17,RPS23,RPS24,RPS26,RPS5,RPS7	5.57e-17
HSA-6791226	Major pathway of rRNA processing in the nucleolus and cytosol	RPL18A,RPL19,RPL27,RPL28,RPL29,RPL31,RPL37A,RPL38,RPS10,RPS17,RPS23,RPS24,RPS26,RPS5,RPS7,XRN2	1.30e-15
HSA-71291	Metabolism of amino acids and derivatives	ACAT1,AHCY,GLS,GOT2,RPL18A,RPL19,RPL27,RPL28,RPL29,RPL31,RPL37A,RPL38,RPS10,RPS17,RPS23,RPS24,RPS26,RPS5,RPS7	7.98e-15
HSA-9010553	Regulation of expression of SLITs and ROBOs	RPL18A,RPL19,RPL27,RPL28,RPL29,RPL31,RPL37A,RPL38,RPS10,RPS17,RPS23,RPS24,RPS26,RPS5,RPS7	7.98e-15
HSA-1430728	Metabolism	ACAT1,ACO2,AHCY,ALDOA,CS,ECHS1,FASN,FDXR,FH,GLS,GOT2,HADHA,HADHB,LDHA,MT-CO2,NDUFS3,PGK1,RPL18A,RPL19,RPL27,RPL28,RPL29,RPL31,RPL37A,RPL38,RPS10,RPS17,RPS23,RPS24,RPS26,RPS5,RPS7,SLC25A6,SLC2A3,SUCLA2,UQCRC1,UQCRC2	8.60e-15
HSA-376176	Signaling by ROBO receptors	PFN1,RPL18A,RPL19,RPL27,RPL28,RPL29,RPL31,RPL37A,RPL38,RPS10,RPS17,RPS23,RPS24,RPS26,RPS5,RPS7	1.05e-14
HSA-5663205	Infectious disease	CTNNB1,GRSF1,RPL18A,RPL19,RPL27,RPL28,RPL29,RPL31,RPL37A,RPL38,RPS10,RPS17,RPS23,RPS24,RPS26,RPS5,RPS7,SLC25A6	1.61e-13
HSA-8953854	Metabolism of RNA	EIF4A1,EIF4B,HNRNPA0,PCBP1,RPL18A,RPL19,RPL27,RPL28,RPL29,RPL31,RPL37A,RPL38,RPS10,RPS17,RPS23,RPS24,RPS26,RPS5,RPS7,SET,XRN2	2.53e-12
HSA-72649	Translation initiation complex formation	EIF4A1,EIF4B,RPS10,RPS17,RPS23,RPS24,RPS26,RPS5,RPS7	4.94e-11
HSA-72702	Ribosomal scanning and start codon recognition	EIF4A1,EIF4B,RPS10,RPS17,RPS23,RPS24,RPS26,RPS5,RPS7	4.94e-11
HSA-422475	Axon guidance	EPHA2,ITGA2,PFN1,RPL18A,RPL19,RPL27,RPL28,RPL29,RPL31,RPL37A,RPL38,RPS10,RPS17,RPS23,RPS24,RPS26,RPS5,RPS7	8.84e-11
HSA-392499	Metabolism of proteins	CCT2,CCT5,CCT6A,CTNNB1,EIF4A1,EIF4B,ERO1L,HIST2H2AB,IARS2,PLAUR,RPL18A,RPL19,RPL27,RPL28,RPL29,RPL31,RPL37A,RPL38,RPS10,RPS17,RPS23,RPS24,RPS26,RPS5,RPS7,SRP14,TUFM,XRN2	1.86e-08
HSA-72695	Formation of the ternary complex, and subsequently, the 43S complex	RPS10,RPS17,RPS23,RPS24,RPS26,RPS5,RPS7	2.59e-08
HSA-1643685	Disease	AHCY,CTNNB1,FDXR,GRSF1,RPL18A,RPL19,RPL27,RPL28,RPL29,RPL31,RPL37A,RPL38,RPS10,RPS17,RPS23,RPS24,RPS26,RPS5,RPS7,SLC25A6	4.26e-08
HSA-1266738	Developmental Biology	CTNNB1,EPHA2,ITGA2,PFN1,RPL18A,RPL19,RPL27,RPL28,RPL29,RPL31,RPL37A,RPL38,RPS10,RPS17,RPS23,RPS24,RPS26,RPS5,RPS7	2.62e-07
HSA-1428517	The citric acid (TCA) cycle and respiratory electron transport	ACO2,CS,FH,LDHA,MT-CO2,NDUFS3,SUCLA2,UQCRC1,UQCRC2	4.70e-07
HSA-71403	Citric acid cycle (TCA cycle)	ACO2,CS,FH,SUCLA2	2.81e-05
HSA-77310	Beta oxidation of lauroyl-CoA to decanoyl-CoA-CoA	ECHS1,HADHA,HADHB	2.81e-05
HSA-77348	Beta oxidation of octanoyl-CoA to hexanoyl-CoA	ECHS1,HADHA,HADHB	2.81e-05
HSA-77350	Beta oxidation of hexanoyl-CoA to butanoyl-CoA	ECHS1,HADHA,HADHB	2.81e-05
HSA-71406	Pyruvate metabolism and Citric Acid (TCA) cycle	ACO2,CS,FH,LDHA,SUCLA2	3.02e-05
HSA-77346	Beta oxidation of decanoyl-CoA to octanoyl-CoA-CoA	ECHS1,HADHA,HADHB	3.72e-05
HSA-390450	Folding of actin by CCT/TriC	CCT2,CCT5,CCT6A	0.00012
HSA-390471	Association of TriC/CCT with target proteins during biosynthesis	CCT2,CCT5,CCT6A,XRN2	0.00017

Supplementary Figure S1. Predicted interactions among proteins deregulated by nitroxoline in our proteomic analysis and proteins previously known to be affected by this drug. The figure shows interactions predicted by STRING analysis among 81 proteins deregulated by nitroxoline treatment and proteins (listed on the right), which were previously suggested to be modulated by this drug[7,9,12-15,17-19].

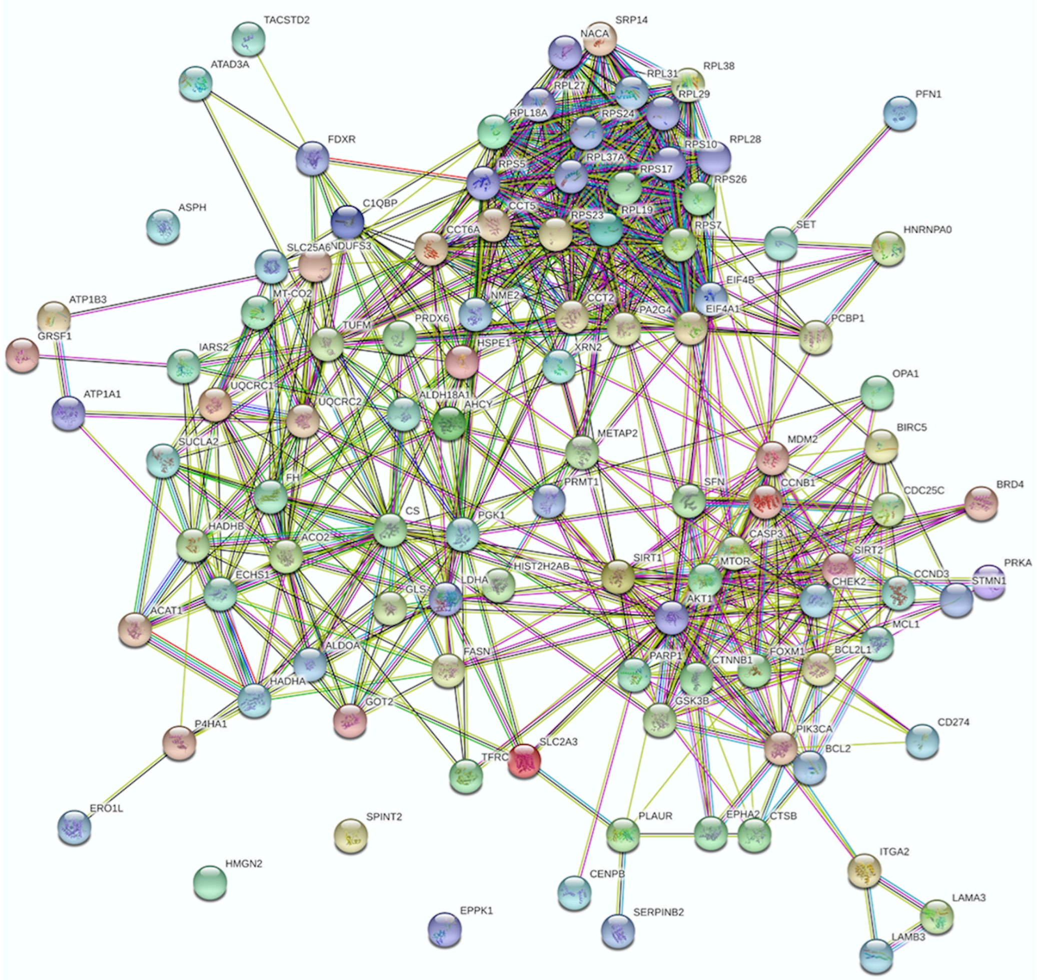

Proteins known to be modulated

SIRT1
SIRT2
METAP2
AMPK
MTOR
CTSB
PI3K
AKT
GSK3B
BCL2
BCLXL
PDL1
MCL1
MDM2
BRD4
PARP1
CASP3
CHK2
FOXM1
SURVIVIN
CENPB
CDC25
CCND3
CCNB1

Supplementary Figure S2. Nitroxoline affects levels of plasma membrane proteins. Downregulation of proteins involved in protein translation and co-translational protein targeting to membrane is expected to affect the levels of plasma membrane proteins. To verify this possibility, we labeled cells with a fluorescent dye that reacts with protein amino groups (L10119, Invitrogen). This dye does not penetrate living cell membranes, where it labels cell surface proteins. The levels of plasma membrane proteins were evaluated after 24 hours of treatment with 27 μM nitroxoline, or vehicle (DMSO). Cells were incubated at room temperature for 30 min in the dark and then washed. For each sample, 10,000 events were recorded. For data analysis a morphological gate was built to select only the population of living cells. The related fluorescent emission was analyzed in terms of mean fluorescence intensity ratio values as described above. Using this assay we observed a significant decrease in cell surface protein labeling after treatment with nitroxoline, as compared to vehicle control. Data shown are the means ± SD of three independent experiments (**$p<0.01$).

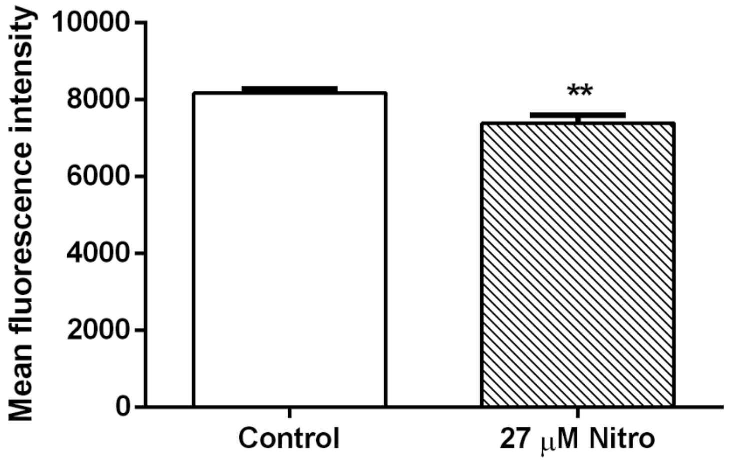

Supplementary Figure S3. AsPC-1 pancreatic cancer cells were treated with nitroxoline, or vehicle (control, CTRL) for 24 and 48 hours. **(A)** Full-length western blots of ATP1B3, β-catenin and β-actin (the corresponding cropped blots are shown in Figure 2A of the main text). Full-length membrane was cut and incubated with the indicated antibodies. β-actin was probed on membrane after stripping of ATP1B3 antibody. **(B)** Full-length western blots of TRFC and β-actin (the corresponding cropped blots are shown in Figure 2A of the main text). Full-length membranes were cut and incubated with the indicated antibodies. The representative β-actin showed in Figure 2A of the main text derives from blot in panel A of this supplementary figure.

A

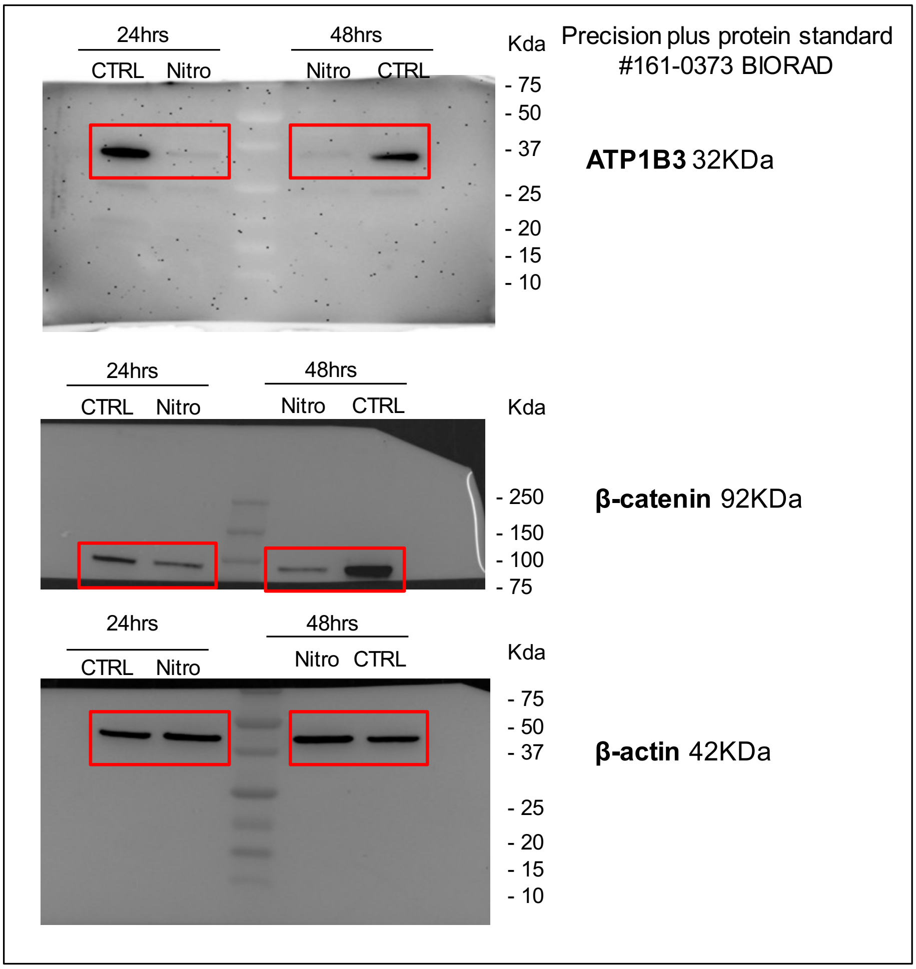

B

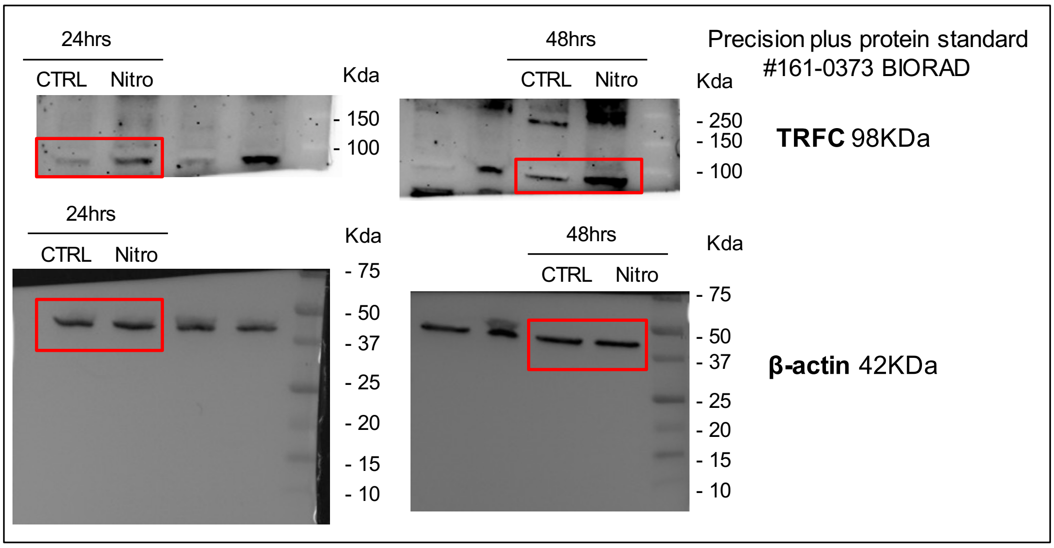

Supplementary Figure S4. AsPC-1 pancreatic cancer cells were treated with nitroxoline, or vehicle (control, CTRL) for 24 and 48 hours. **(A)** Full-length western blots of PI3K and β-actin (the corresponding cropped blots are shown in Figure 2B of the main text). Full-length membranes were cut and incubated with the indicated antibodies. **(B)** Full-length western blots of GSK3β, pSer^{139}H2AX and β-actin (the corresponding cropped blots are shown in Figure 2B and Figure 5B of the main text). The full-length membrane was cut and incubated with the indicated antibodies. pSer^{139}H2AX was probed after cutting the upper portion of the blot used for β-actin detection. **(C)** Full-length western blots of AKT, pSer^{473}AKT, pSer^{9}GSK3β and β-actin (the corresponding cropped blots are shown in Figure 2B of the main text). The full-length membrane was incubated first with the pSer^{473}AKT antibody and reprobed with the pSer^{9}GSK3β, AKT and β-actin antibodies after stripping. The representative β-actin showed in Figure 2B of the main text derives from blot in panel A of this supplementary figure.

A

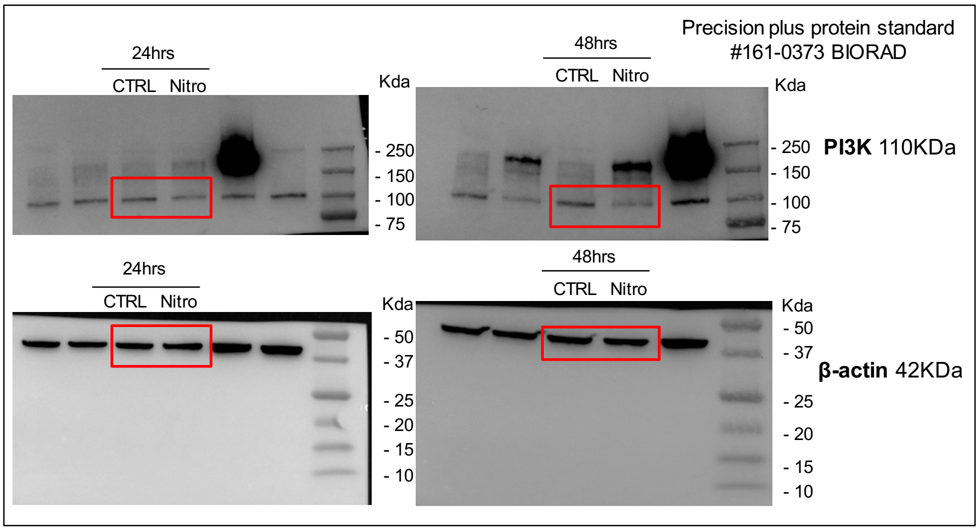

B

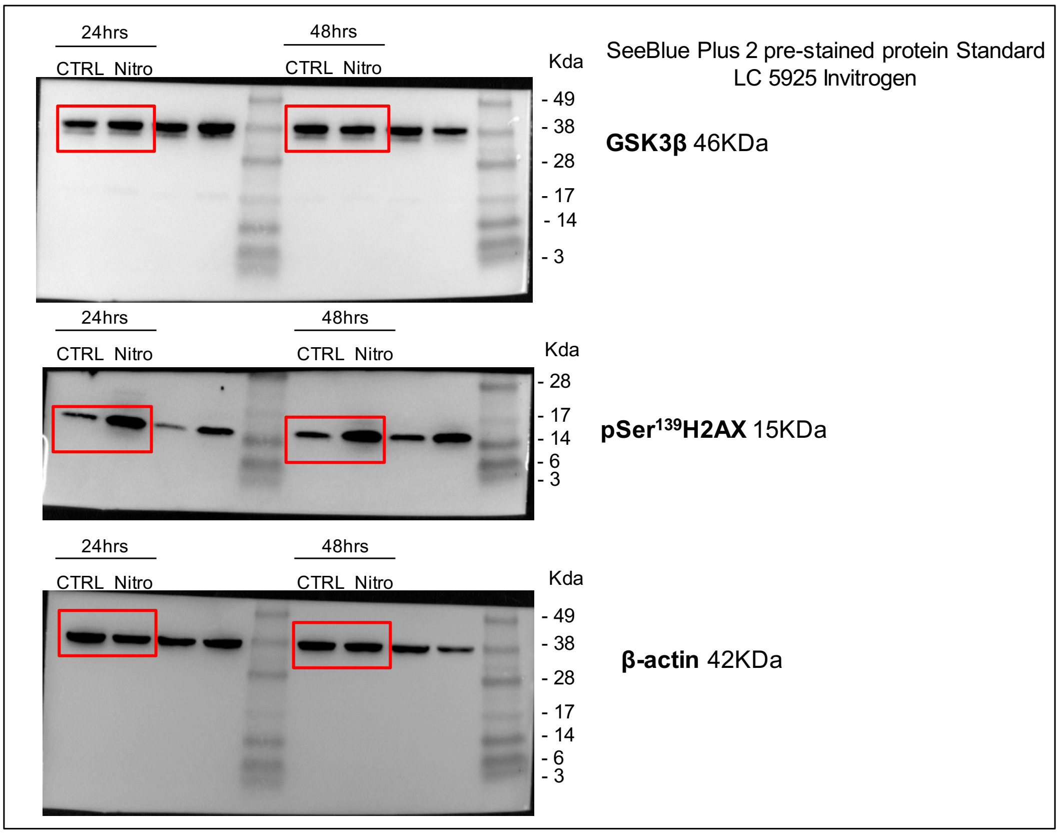

C

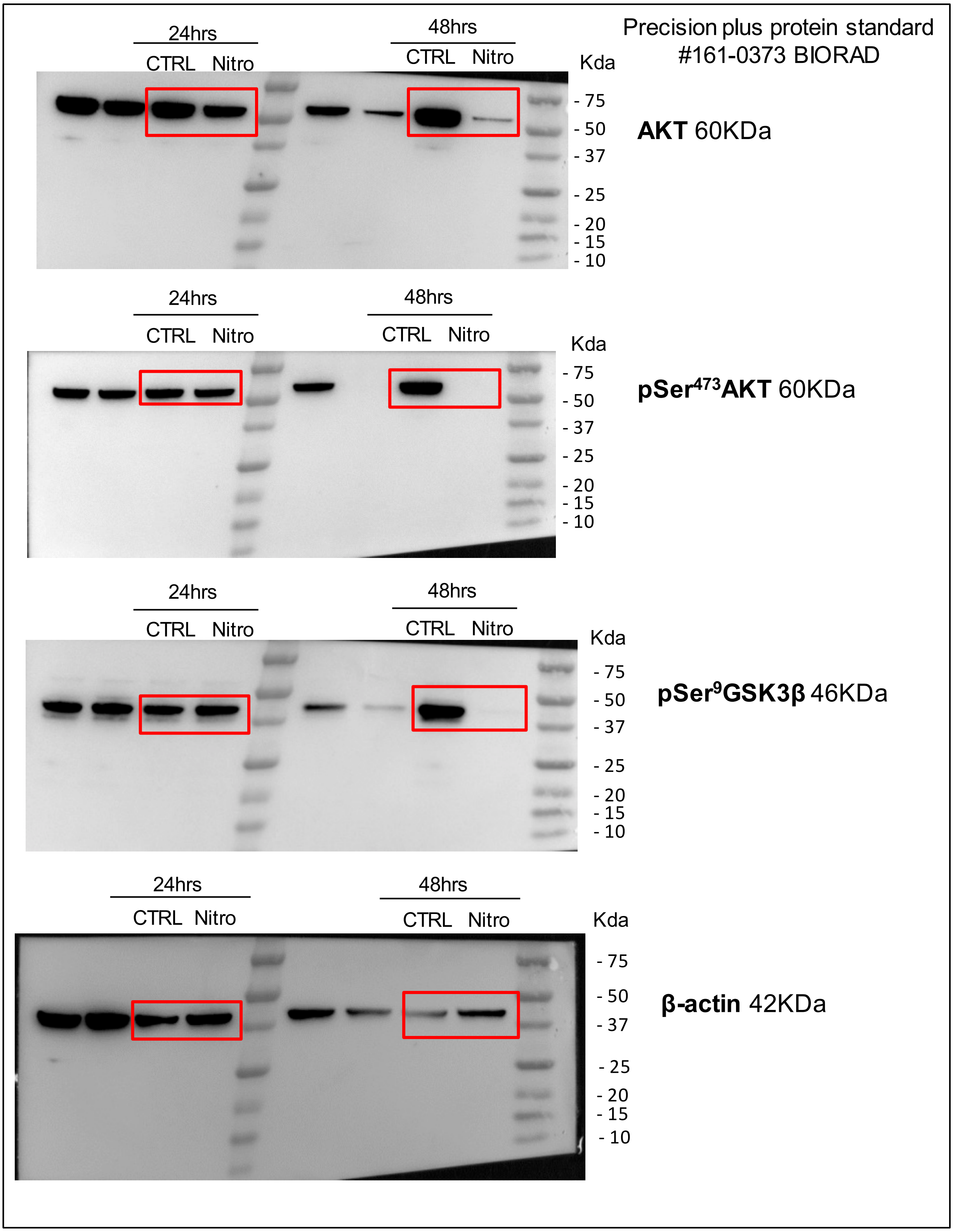
24hrs
48hrs
CTRL
Nitro
Precision plus protein standard
#161-0373 BIORAD
Kda
- 75
- 50
- 37
- 25
- 20
- 15
- 10
AKT 60KDa
pSer473AKT 60KDa
pSer9GSK3β 46KDa
β-actin 42KDa
